# Simultaneous Diagnosis of Myasthenia Gravis and Neurosyphilis, With Symptoms Improving After Neurosyphilis Treatment: A Case Report

**DOI:** 10.7759/cureus.102139

**Published:** 2026-01-23

**Authors:** Juan Fernando Ortiz, Nicholas J Lannen, Maria Daniela Peralta, Camila D Lopez

**Affiliations:** 1 Neurology, Corewell Health Medical Group, Grand Rapids, USA; 2 Neurology, Corewell Health, Grand Rapids, USA; 3 Medicine, Universidad de Cuenca, Cuenca, ECU; 4 Medicine, Universidad San Francisco de Quito, Quito, ECU

**Keywords:** bulbar weakeness, dysarthria, late neurosyphilis, myasthenia gravis, single nerve fiber

## Abstract

Simultaneous diagnosis of myasthenia gravis (MG) and early asymptomatic neurosyphilis is extremely rare. There have only been two cases described in the literature. To the best of our knowledge, this was the first case of MG resolving with concurrent treatment of neurosyphilis.

Our patient was a 67-year-old male who presented to the hospital with a diffuse rash, bilateral ptosis, bilateral facial weakness, and bulbar symptoms (dysarthria, difficulty with mastication, and difficulty drinking fluids).  After testing positive for syphilis in serum and cerebrospinal fluid (CSF), the patient was diagnosed with neurosyphilis. Thus, it was suspected that his neurological findings were manifestations of early symptomatic neurosyphilis. The patient was treated with intravenous penicillin G for one week, and later, it was switched to intravenous ceftriaxone for two weeks due to cost and convenience. All symptoms resolved after three to four weeks. After being discharged, the patient tested positive for acetylcholine receptor antibodies. Subsequently, the patient underwent an electromyogram (EMG), which revealed a decremental response, confirming a diagnosis of MG. Infections are known to trigger autoimmune diseases and can lead to the development of new-onset MG.

Neurosyphilis is relatively highly associated with other central nervous system (CNS) antibodies and can trigger other neurological disorders. Recognizing the differences in presentation and performing an accurate and comprehensive neurological exam can help differentiate early symptomatic neurosyphilis from neuromuscular disorders such as MG.

## Introduction

Myasthenia gravis (MG) is an autoimmune disease that affects neuromuscular junctions, caused by autoantibodies targeting postsynaptic acetylcholine receptors (AChR) at the neuromuscular junction, resulting in impaired neuromuscular transmission [1]. The clinical features of myasthenia gravis include ptosis, diplopia, bulbar symptoms, fatigue, and weakness, which are typically proximal and descending, worsen with repetitive activity, and improve with rest [1].

Diagnosis is typically confirmed via clinical assessment and serological assays for specific antibodies, such as acetylcholine receptor (AChR) antibodies (detected in about 85% of generalized myasthenia gravis cases), muscle-specific kinase (MuSK) antibodies in roughly 6% of cases, and low-density lipoprotein receptor-related protein 4 (LRP4) antibodies in approximately 2% of cases [2]. Electrodiagnostic evaluations, including repetitive nerve stimulation (RNS), reveal a decremental response, whereas single-fiber electromyography (SFEMG) exhibits elevated jitter and potential blocking, establishing it as the most sensitive test for MG [1]. 

Neurosyphilis, resulting from invasion of the central nervous system (CNS) by *Treponema pallidum*, is a serious and often overlooked complication of untreated syphilis [3,4]. It can present asymptomatically or symptomatically [3,4]. In addition, neurosyphilis can be classified into different stages, such as early, intermediate, and late manifestations. Early forms typically affect the cerebrospinal fluid (CSF), meninges, and cerebral vessels, causing syphilitic meningitis and meningovascular disease, while late forms more often involve the brain and spinal cord parenchyma. Diagnosis uses a positive CSF-venereal disease research laboratory (VDRL) (highly specific but insensitive) or clinical signs with CSF pleocytosis, and elevated protein [3,4]. Notably, neurosyphilis shares significant symptomatic overlap with MG, particularly in bulbar and ocular features.

The coexistence of MG and neurosyphilis is exceedingly rare, with only two prior reports in the literature describing concurrent diagnoses [5, 6]. Infections, including syphilis, have been implicated in triggering or exacerbating autoimmune conditions like MG. However, to the best of our knowledge, there are no prior cases that have documented resolution of MG symptoms solely following neurosyphilis treatment without adjunctive MG-specific therapies.

Herein, we report a unique case of a 67-year-old man with simultaneous diagnoses of early asymptomatic neurosyphilis and seropositive MG, where initial neurological symptoms attributed to neurosyphilis fully resolved after antibiotic therapy alone, with MG confirmed post-treatment via AChR antibodies and SFEMG. This case highlights the diagnostic challenges arising from overlapping syndromes, the potential role of infectious triggers in MG pathogenesis, and the critical importance of differentiating between neurosyphilis across all stages and MG to ensure targeted therapy, minimize treatment delays, and avoid misdiagnosis.

## Case presentation

The patient is a 67-year-old male with a medical history of hypertension, type 2 diabetes, and sleep apnea, with no family history of neuromuscular disorders. The patient gave written and oral consent for the images and this report. 

He arrived at the hospital with complaints of bilateral ptosis, bilateral facial weakness, and bulbar symptoms, including dysarthria and difficulty with mastication. He reported having slurred speech and trouble with chewing, especially from his right side, as well as droopy eyelids on both sides 48 hours before coming to the hospital, and facial drooping, more prominent on the right side, 24 hours before coming to the hospital. His coworkers noticed the facial droop and urged the patient to seek urgent medical attention. At the emergency department, the patient arrived stable, breathing on room air without any respiratory distress. Upon further questioning, the patient reported feeling easily fatigued for the past month. He denied experiencing double vision, respiratory distress, weakness, sensory symptoms, or difficulty walking, and there have been no recent changes in medication or travel history. In terms of his social history, the patient is a man who has sex with other men. His last sexual encounter was four months before admission.

**Figure 1:**
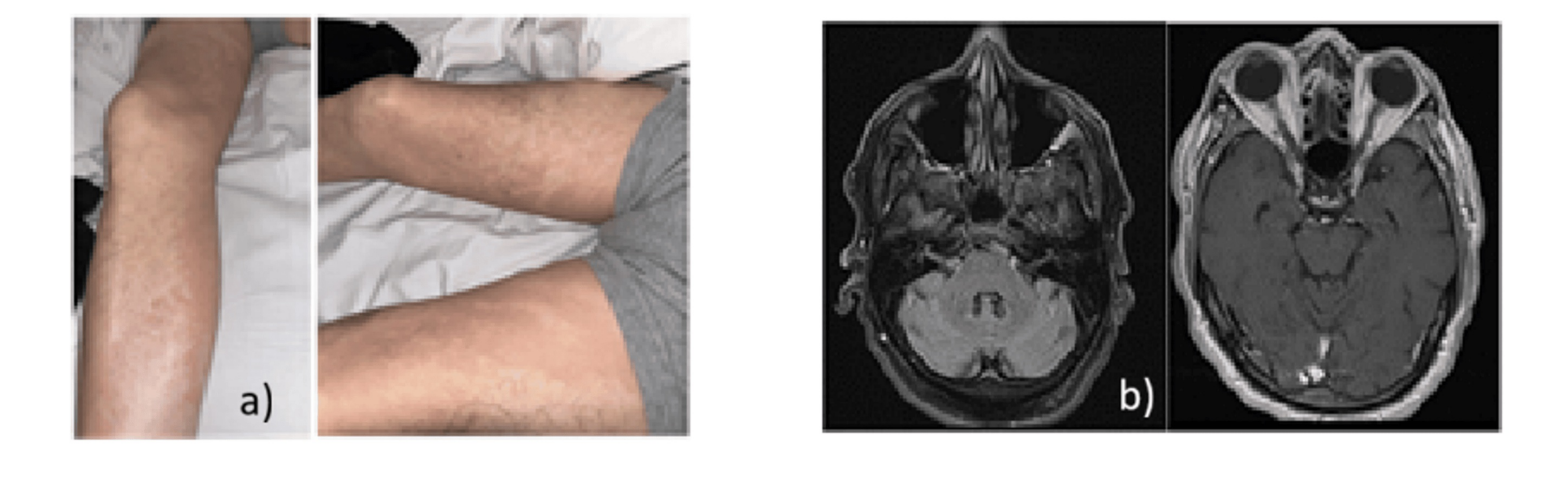
Erythematous rash in the lower extremities (a) and MRI T2 FLAIR (left) and T1 (right) (b) The patient exhibits an erythematous rash, which is attributed to syphilis (interestingly, there was no rash on the palms of the hands). The brain MRI did not show any cranial nerve or meningeal enhancement. FLAIR: fluid-attenuated inversion recovery

Upon examination, the patient was fully oriented but had moderate dysarthria and no aphasia. His gaze was midline, with more pronounced ptosis in the right eye than the left. Ptosis was slightly fatigable with upgaze without causing double vision. The patient exhibited bilateral facial weakness, which was more pronounced on the right side, in a lower motor pattern. According to the Medical Research Council [7], muscle strength was graded 5/5 in both the upper and lower extremities. The sensation of pinprick, vibration, and proprioception was normal. Reflexes were +1 throughout. The patient had an erythematous rash on both legs. Head and neck computed tomography angiography (CTA), as well as brain MRI with and without contrast, were unremarkable. Figure 1a and 1b show the patient's rash and his MRI findings.

The patient's initial labs revealed increased inflammatory markers and positive rapid plasma reagin (RPR), which, in conjunction with the rash, were indicative of syphilis. In light of the bulbar symptoms, a myasthenia panel and a lumbar puncture were ordered. Table 1 shows laboratory results and CSF analysis. 

**Table 1:** Summary of laboratory and cerebrospinal fluid labs. The patient's serum antibodies tested positive for syphilis. In addition, he was diagnosed with early neurosyphilis, which can be diagnosed if there is a positive VDRL on CSF, high protein >45 mg/dL, and >5 cells/μL. ESR: erythrocyte sedimentation rate, RPR: rapid plasma reagin, ANA: antinuclear antibody, VDRL: venereal disease research laboratory, mg: milligrams, ug: micrograms, L: Liters, dL: deciliter, μL: microliters MuSK: muscle-specific kinase, ACh: acetylcholine, Ab: antibody

Test	Observed Value	Reference Range
C-reactive protein (mg/L)	28.2	< 10
ESR	45 (mm/hr)	< 20
Hemoglobin	9.5 (g/dL)	12–16 (female) / 13–17 (male)
Haptoglobin	293 (mg/dL)	30–200
RPR	Positive, 1:64 titer	Nonreactive
Total antibody (treponemal test)	Positive	Negative
HIV	Negative	Negative
ANA	Negative	Negative
Lyme antibody	Negative	Negative
CSF		
Cell count	72 (cells/μL)	< 5
Lymphocytes	33 (cells/μL)	0–6
Glucose	111 (mg/dL)	40–70
Protein	105 (mg/dL)	15–45
VDRL	Positive	Nonreactive
Myasthenia/Eaton Lambert panel		
P/Q-Type Calcium Channel Ab	0.00	<=0.02 nmol/L
ACh Receptor (Muscle) Binding Ab	0.33	<=0.02 nmol/L
MuSK Ab	0.00	<=0.02 nmol/L

Given the positive VDRL on CSF, elevated CSF protein, and lymphocytic pleocytosis, the patient was diagnosed with neurosyphilis; his neurological symptoms were attributed to early symptomatic neurosyphilis. He was treated with intravenous penicillin G for one week and was later switched to intravenous ceftriaxone. The patients' facial weakness, ptosis, and difficulty swallowing improved rapidly. 

One week after discharge, the myasthenia/Eaton‑Lambert panel returned: acetylcholine receptor binding antibody was positive at 0.33 nmol/L; P/Q and MuSK antibodies were undetectable. Neurology contacted the patient and arranged follow‑up; at that time, he reported no further symptoms. Two to three days after discharge, he was seen in the clinic and had a normal examination without ptosis, facial weakness, or proximal weakness.

Four months after symptom onset, the patient underwent electrodiagnostic testing while asymptomatic. Nerve conduction studies demonstrated normal sensory nerve action potentials (SNAPs) and compound muscle action potentials (CMAPs)with no evidence of axonal or demyelinating damage. Repetitive nerve stimulation (RNS) (3 Hz) of the right ulnar, spinal accessory, and facial nerves showed no decremental response. SFEMG was performed on the frontalis, which demonstrated increased jitter without blocking: the overall mean consecutive difference (MCD) was 72.6 μs, and five of 21 fiber pairs had MCDs greater than 74.1 μs (laboratory normal for frontalis approximately <35-40 μs). No blocking was observed. These SFEMG findings, together with a positive acetylcholine receptor binding antibody, supported the diagnosis of MG (see Appendices).

At this point, in retrospect, it was concluded that the patient's initial symptoms were unlikely to be related to neurosyphilis. The patient most likely had early asymptomatic neurosyphilis simultaneously with MG. One year after the onset of symptoms, the patient remains asymptomatic. His last RPR qualitative study was non-reactive. 

## Discussion

We present a case of neurosyphilis and a simultaneous diagnosis of MG. MG coexisting with neurosyphilis has been documented only twice before [5, 6]. In 1993, Wüllenveber et al. described a 38-year-old male with HIV who had neurosyphilis and MG along with a thymoma. The patient's symptoms improved after treating syphilis with aqueous crystalline penicillin treatment, but they were not entirely resolved. He only became entirely symptom-free after the thymoma was removed [5]. Borges et al. briefly described a case series in which one patient diagnosed with MG had a myasthenic crisis precipitated by syphilis infection and the development of syphilitic meningitis. The patient's symptoms partially improved with antibiotic therapy [6]. This is the first instance, to our knowledge, in which the symptoms of MG were completely resolved by treating neurosyphilis infection without immunotherapy or acetylcholinesterase inhibitors.

Syphilis is known as "the great imitator" owing to its broad spectrum of clinical symptoms [8]. For neurosyphilis diagnosis, a positive VDRL in the cerebrospinal fluid acts as a vital marker (highly specific), yet it has limited sensitivity. Consequently, when the test is negative, check for other CSF irregularities in patients with confirmed syphilis, like lymphocytic pleocytosis and elevated protein levels, to confirm the diagnosis [3,4].

Table 2 shows the timing, diagnostic testing, and clinical features of early symptomatic syphilis and early symptomatic neurosyphilis [3,4].

**Table 2:** Clinical features of early asymptomatic and symptomatic neurosyphilis. In our case, our patient met the criteria for asymptomatic neurosyphilis. VDLR: Venereal Disease Research Laboratory, CSF: Cerebrospinal Fluid

Type of Neurosyphilis	Timing After Infection	Diagnostic Criteria / Clinical Features
Early Asymptomatic Neurosyphilis	Weeks to up to a year after infection.	Defined by positive VDLR on CSF, or elevated cerebrospinal fluid (CSF) protein (>45 mg/dL), and lymphocytic pleocytosis (>5 white blood cells/cubic millimeter CSF) in the absence of other known causes of these abnormalities
			Early Symptomatic Neurosyphilis	Usually develops within 12 months of infection	Clinical features include meningitis, usually basilar meningitis. Complications include multiple cranial nerve palsies (specifically III, VI, VII, VIII) and hydrocephalus leading to increased intracranial pressure.

In retrospect, the patient's symptoms, such as ptosis, dysphagia, dysarthria, and bilateral facial weakness, were unlikely to be secondary to neurosyphilis. For example, facial weakness in neurosyphilis typically involves unilateral cranial nerve involvement, in contrast to MG, where it tends to be bilateral [8]. Bulbar symptoms may arise from involvement of cranial nerves IX or X, but these nerves are rarely affected in neurosyphilis. According to a comparative study of HIV-positive and HIV-negative patients with neurosyphilis, the most commonly involved cranial nerves were VIII (76%), V (19%), VII (10%), III, and XII (5% each) [9]. 

Furthermore, when facial weakness or bulbar symptoms do occur in neurosyphilis, they are usually in the context of meningitis, as the condition affects the basilar meninges, causing inflammation, altered mental status that eventually impacts the cranial nerves [4]. When facial weakness occurs outside of meningitis, it is more often associated with stroke-like events due to arteritis affecting small vessels (Nissl-Alzheimer arteritis) or medium and large arteries (Heubner arteritis) within the central nervous system [4]. In addition, ptosis would be a very uncommon symptom of neurosyphilis. 

The justification that syphilis might trigger MG can be justified as infections are a well-known trigger for myasthenia. According to Barzago et al., a study with a molecular signature of RNA transcript suggested that 17% of MG cases may have been triggered by infection [9]. Infections associated with MG, including hepatitis B, hepatitis C, herpes simplex virus (HSV), and HIV, have been linked to Zika virus and West Nile, among others [10-12]. Furthermore, other neuroinvasive infections, such as neuroinvasive West Nile virus, have been linked not only with triggering MG but also other autoimmune conditions such as Guillain-Barré Syndrome (GBS) and Stiff Person Syndrome [11]. Among the suggested mechanisms for this relationship are molecular mimicry, increased inflammatory response, and autoreactivity of B and T lymphocytes [12]. 

In our case, it could be argued that MG was a response to an autoimmune process secondary to neurosyphilis. Neurosyphilis has been linked to autoimmune conditions such as leucine-rich glioma inactivated 1 (LGI) encephalitis, anti-N-methyl-D-aspartate (NMDA) receptor encephalitis, gamma-aminobutyric acid (GABA) B encephalitis, and neuromyelitis optica spectrum disorder (NMOSD) [13-15]. A retrospective study of 81 neurosyphilis patients revealed a relatively high prevalence of autoimmune diseases [16]. Three cases (3.7%) tested positive for specific antibodies (anti-aquaporin-4 antibody, anti-glutamic acid decarboxylase 65 antibody (GAD-65), anti-NMDA receptor antibody)[16]. It has been suggested that syphilis may cause secondary immune impairment, but the mechanism is not fully understood [16]. Possible mechanisms include cross-reactions and alterations in the normal immune tolerance [16].

## Conclusions

The simultaneous diagnosis of neurosyphilis and MG is exceedingly rare, with only three cases documented in the literature, including our own. Infectious diseases frequently serve as triggers for exacerbations or the onset of new MG. In this instance, the emergence of MG in the context of neurosyphilis may be linked to the heightened prevalence of concomitant autoimmune disorders in patients with neurosyphilis. Despite their overlapping symptoms, a thorough clinical evaluation, combined with a clear understanding of the key distinctions between MG and early symptomatic neurosyphilis, can help prevent initial misdiagnoses in future cases.

## Figures and Tables

**Figure 2:**
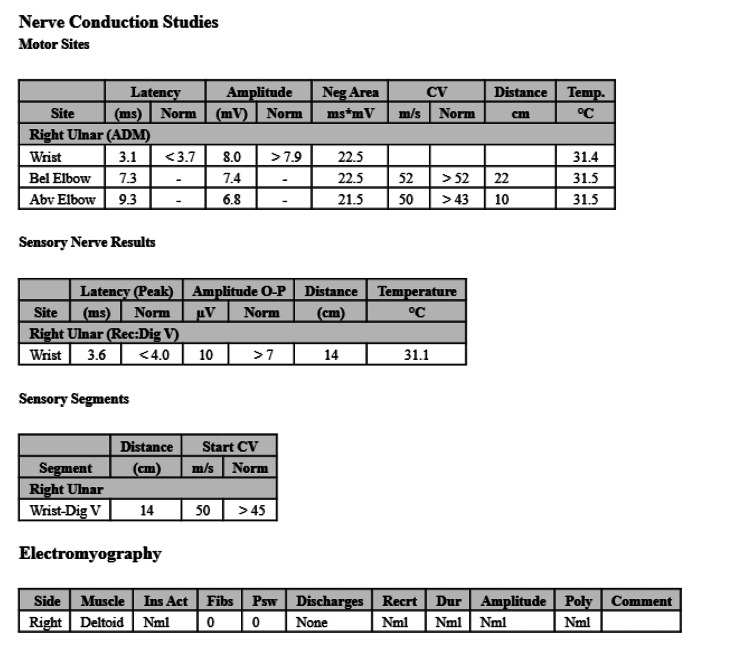
Nerve conduction studies and elctromyography Nerve conduction studies included normal right ulnar sensory and motor responses. Electromyography was normal ms: Milliseconds, Norm: Normal, mV: Millivolts, Neg Area: Negative Area, ms*mV: Millisecond-millivolts, CV: Conduction Velocity, m/s: Meters per second, cm: Centimeters, ADM: Abductor Digiti Minimi, Abv Elbow: Above Elbow, O-P: Onset to Peak, μV: Microvolts, Rec:Dig V: Recorded at Digit V, Ins Act: Insertional Activity, Fibs: Fibrillations, Psw: Positive Sharp Waves, Rect: Recruitment, Dur: Duration, Ampl: Amplitude, Poly: Polyphasic, Nml: Normal

**Figure 3:**
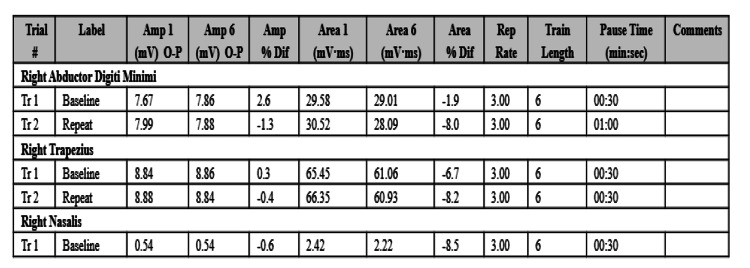
RNS (3 Hz) of three muscles (right abductor digiti minimi, right trapezius, right nasalis) Tr: Trial, Amp: Amplitude, O-P: Onset to Peak, % Dif: Percent Difference, Area: Area, mV: Millivolt, ms: Millisecond, Rep Rate: Repetition Rate, Train Length: Train Length, Pause Time: Pause Time, ADM: Abductor Digiti Minimi, RNS: Repetitive Nerve Stimulation, CMAP: Compound Muscle Action Potential

## Appendices

Figure 2 shows the nerve conduction studies and electromyography of the patient.

Figure 3 shows the repetitive nerve stimulation test.

Table 3 shows the single-fiber electromygraphy (SFEMG) of the right frontalis muscle, which revealed a mean consecutive difference (MCD) of 72.6 μsec with a standard deviation (SD) of 66.6 μs. Five of 21 fiber pairs demonstrated an MCD greater than 74.1 μsec, consistent with increased jitter. The mean jitter was 70.7 μs (SD of 66.8 μs). No blocking was observed (0%).

**Table 3:** SFEMG of the right frontalis muscle revealed a MCD of 72.6 μsec, with five of 21 pairs exhibiting a MCD greater than 74.1 μsec, indicative of increased jitter. IPI: interpotential interval, µs: microseconds, MCD: mean consecutive difference, MSD: mean sorted difference, Freq: frequency, Hz: hertz, SFEMG: single-fiber electromyography

Right Frontalis							
Run	Samples	Blocks	IPI [µs]	Jitter [µs]	MCD [µs]	MSD [µs]	Freq [Hz]
1.1	30	No	806	228	228	269	37,3
2.1	40	No	969	257	257	303	11,9
3.1	30	No	477	36,2	36,2	38,6	9,53
3.2	37	No	2291	18,4	18,4	44,2	9,53
4.1	97	No	458	54,3	60,1	54,3	13,1
5.1	29	No	328	23,7	30,6	23,7	33,8
6.2	85	No	904	35,4	35,4	40,2	14,7
6.3	94	No	390	23,4	23,4	34,7	14,7
6.4	87	No	476	22	22	23,9	14,7
7.1	52	No	5780	42,2	42,2	49,1	28,2
7.3	58	No	445	38,2	38,2	40	28,2
8.1	43	No	1876	35,2	35,2	37,6	34,5
8.2	25	No	1659	47,4	47,4	58,4	34,5
8.3	18	No	2689	48,4	61,1	48,4	34,5
9.1	37	No	466	131	134	131	16,2
9.3	60	No	2862	50,4	52,5	50,4	16,2
10.1	75	No	2809	34,8	37,5	34,8	21,1
11.1	47	No	470	55,6	55,7	55,6	22,8
11.2	44	No	485	125	130	125	22,8
12.1	86	No	415	43	44	43	11,6
12.2	85	No	624	136	136	147	11,6

The SFEMG test on the right frontalis muscle shows positive for myasthenia gravis due to significantly elevated jitter values (MCD often >50 µs in multiple runs), indicating impaired neuromuscular transmission at the junction. Normal jitter is typically <34-44 µs mean, with <10% of pairs abnormal; here, over half the 21 runs exceed thresholds, with outliers up to 257 µs, without blocking. This pattern is highly specific for MG, especially when routine NCS/EMG are normal, confirming autoimmune disruption of acetylcholine receptors.
